# Supplementary material for: Identification of residue pairing in interacting β-strands from a predicted residue contact map
Source: BMC Bioinformatics. 2018 Apr 19;19:146. doi: 10.1186/s12859-018-2150-1 (PMC5907701; doi:10.1186/s12859-018-2150-1)
Supplement: Supplementary file 1 — Text S1. Technical details of the γ-normalized scale method for ridge detection; Table S1. List of domains in the training set; Table S2. Results of structure prediction for 61 mainly β proteins. (PDF 537 kb) [file 12859_2018_2150_MOESM1_ESM.pdf]

## Text S1. Technical details of the γ-normalized scale method for ridge detection.

***Estimate of the gradient and Hessian matrix***

For each given point on the contact map, we focus on the extended surrounding region with the size of 5×5. For all 25 points, we estimate a quadratic function via ordinary least squares. Let us use $\Delta x$ and $\Delta y$ to denote the distances from the given point. On the boundary of the matrix, the value on the edge is repeated to extend the contact map for analysis:

$$\text{Score} = A + B\Delta x + C\Delta y + \frac{1}{2}D\Delta x^2 + \frac{1}{2}E\Delta y^2 + F\Delta x\Delta y .$$

Hence, we could get the gradient **∇f** and Hessian matrix **H** as follows,

$$\nabla \mathbf{f} = \begin{bmatrix} B \\ C \end{bmatrix}$$

$$\mathbf{H} = \begin{bmatrix} D & F \\ F & E \end{bmatrix} .$$

After the eigendecomposition, we could get the eigenvalues from the Hessian matrix:

$$\lambda = \frac{D + E \pm \sqrt{(D\text{-}E)^2 + 4F^2}}{2} .$$

In this case, the *NL* could be simplified as,

$$NL = (D + E)^2 \left( (D - E)^2 + 4F^2 \right) .$$

***The γ-normalized scale method***

The γ-normalized scale method is introduced in Lindeberg's work [1]. Here, we describe the procedure briefly.

Firstly, we define an additional scale dimension to the existing 2D contact map. The scale dimension represents the convolution of the original contact map with a Gaussian smooth filter of scale $\sigma$. A Gaussian filter is defined as:

$$g(\sigma) = \frac{1}{2\pi\sigma^2} e^{-\frac{x^2+y^2}{2\sigma^2}} .$$

The *NL* ridge strength could be calculated for each smoothed map at any given point. So we could define the *NL* as a 3 dimension function of $x$, $y$ and $\sigma$. In order to depict more details of the ridge, we define the $NL_\gamma$ as $NL_\gamma = \sigma^\gamma \left( \lambda_p^2 - \lambda_q^2 \right)^2$ . We want to use a carefully-selected $\gamma$ to normalize the *NL*. The purpose is to make the $NL_\gamma$ achieve the maxima at the ridge width of $\sigma_0$.

In order to determine the unknown variable $\gamma$, we assume that there is a Gaussian ridge parallel to the y-axis. The ridge has a height $A$ and a Gaussian standard deviation $\sigma_0$:

$$f(x, y) = Ae^{-\frac{x^2}{2\sigma_0^2}} .$$

When applying with a Gaussian filter, we obtained the 3-dimensional function F:

$$F(x, y, \sigma) = f \circ g(\sigma) = \frac{\sigma_0 A}{\sqrt{\sigma_0^2 + \sigma^2}} e^{-\frac{x^2}{2\left(\sigma_0^2 + \sigma^2\right)}}.$$

The Hessian matrix of F is

$$\mathbf{H}(F) = \mathbf{H}\left( \frac{\sigma_0 A}{\sqrt{\sigma_0^2 + \sigma^2}} e^{-\frac{x^2}{2\left(\sigma_0^2 + \sigma^2\right)}} \right)$$

$$= \begin{bmatrix} \frac{\left(x^2 - \sigma_0^2 - \sigma^2\right)\sigma_0 A}{\left(\sigma_0^2 + \sigma^2\right)^{\frac{5}{2}}} e^{-\frac{x^2}{2\left(\sigma_0^2 + \sigma^2\right)}} & 0 \\ 0 & 0 \end{bmatrix} = \begin{bmatrix} \lambda_p & 0 \\ 0 & \lambda_q \end{bmatrix}.$$

On the ridge x=0

$$\mathbf{H}(F)\big|_{x=0} = \begin{bmatrix} \frac{\sigma_0 A}{\left(\sigma_0^2 + \sigma^2\right)^{\frac{3}{2}}} & 0 \\ 0 & 0 \end{bmatrix} = \begin{bmatrix} \lambda_p & 0 \\ 0 & \lambda_q \end{bmatrix}$$

$$NL_\gamma = \sigma^\gamma \left(\lambda_p^2 - \lambda_q^2\right)^2 = \sigma^\gamma \left( \frac{\sigma_0 A}{\left(\sigma_0^2 + \sigma^2\right)^{\frac{3}{2}}} \right)^4 = \sigma^\gamma \frac{\sigma_0^4 A^4}{\left(\sigma_0^2 + \sigma^2\right)^6}.$$

$$\frac{\partial NL_\gamma}{\partial \sigma} = \frac{\sigma^{\gamma\text{-}1}\left(\gamma\sigma_0^2 + (\gamma\text{-}12)\sigma^2\right)\sigma_0^4 A^4}{\left(\sigma_0^2 + \sigma^2\right)^7}$$

We want to adjust $\gamma$ to maximize $NL_\gamma$ at $\sigma = \sigma_0$,

$$\frac{\partial NL_\gamma}{\partial \sigma}\bigg|_{\sigma=\sigma_0} = \frac{\sigma^{\gamma\text{-}1}\left(\gamma\sigma_0^2 + (\gamma\text{-}12)\sigma^2\right)\sigma_0^4 A^4}{\left(\sigma_0^2 + \sigma^2\right)^7}\bigg|_{\sigma=\sigma_0} = \frac{1}{64}\sigma_0^{\gamma\text{-}2} A^4 (\gamma - 6) = 0.$$

$$\Rightarrow \gamma = 6$$

Thus, the $NL_\gamma$ will achieve maximal when the scale dimension equals the ridge width, if we set $\gamma$=6. We further defined the height of the ridge:

$$NL_\gamma\big|_{\sigma=\sigma_0} = \sigma^6 \frac{\sigma_0^4 A^4}{\left(\sigma_0^2 + \sigma^2\right)^6}\bigg|_{\sigma=\sigma_0} = \frac{A^4}{64\sigma_0^2}.$$

$$\Rightarrow A = \sqrt[4]{\max(NL_\gamma) \times 64 \times \sigma_{\max}^2}$$

In practice, we smooth the map with a series of scales from 1 to 3 using a step size of 0.1. As shown in the following figure, the smoothed map with a scale less than 1 has almost no effect because of the discrete properties of map and Gaussian filter. The scale will finally represent the ridge width, and a scale value of larger than 3 means that the captured ridge is too thick to be a β-β contact pattern.

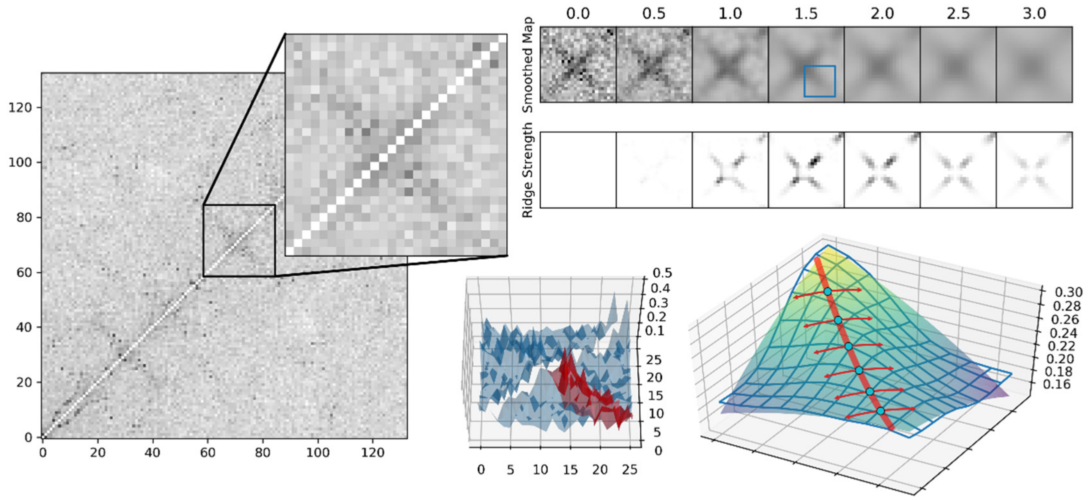


**The demonstration of ridge calculation.** We smooth the contact map with different scales. The smoothed map and the corresponding ridge strength are displayed. 3D figures in the lower right corner are enlarged representation of the signals and identified ridges in the blue square shown in one of the smoothed 2D maps. The data in this figure were generated from the protein 1AHQA.

**Table S1. List of domains in the training set.**

| CATH Domain | *L* | *N* | In shrunk set | CATH Domain | *L* | *N* | In shrunk set |
|---|---|---|---|---|---|---|---|
| 1a31A03 | 150 | 1271 | √ | 2hnuA00 | 81 | 153 | √ |
| 1af6A00 | 421 | 632 | √ | 2hr7A02 | 118 | 2261 | √ |
| 1amuA03 | 81 | 25945 | √ | 2i9dA00 | 213 | 4631 | |
| 1at0A00 | 145 | 1289 | | 2iahA02 | 134 | 20793 | √ |
| 1avvA00 | 99 | 861 | √ | 2iayA00 | 114 | 227 | |
| 1b25A01 | 209 | 1540 | √ | 2icgA00 | 159 | 2614 | |
| 1b4uB00 | 298 | 4909 | √ | 2ichB02 | 131 | 860 | |
| 1bcpB01 | 86 | 7 | √ | 2idbC02 | 145 | 1827 | |
| 1c3kA00 | 143 | 2163 | √ | 2ii3A01 | 224 | 6479 | √ |
| 1c4kA04 | 129 | 2196 | √ | 2im5A00 | 389 | 3444 | √ |
| 1c4zA02 | 81 | 3280 | √ | 2in0A00 | 139 | 2248 | √ |
| 1c4zA03 | 109 | 3415 | √ | 2isbA00 | 184 | 1542 | |
| 1c96A01 | 201 | 5003 | √ | 2it9D00 | 121 | 123 | |
| 1c96A02 | 113 | 7476 | √ | 2iv2X03 | 195 | 12746 | |
| 1c96A03 | 175 | 6514 | √ | 2j58A03 | 81 | 4360 | |
| 1ccwB02 | 66 | 143 | √ | 2j8gA02 | 82 | 6202 | √ |
| 1ciyA02 | 196 | 206 | √ | 2j8gA03 | 58 | 5072 | √ |
| 1cksB00 | 78 | 399 | √ | 2jkbA03 | 98 | 37 | √ |
| 1cseI00 | 63 | 1168 | √ | 2nmlA00 | 100 | 188 | √ |
| 1divA02 | 90 | 3675 | √ | 2nvnA00 | 120 | 126 | |
| 1dkgB02 | 57 | 4449 | √ | 2o01D01 | 95 | 117 | √ |
| 1dl5A02 | 116 | 5 | √ | 2o34A00 | 249 | 1362 | |
| 1dt9A01 | 105 | 372 | √ | 2ogkD00 | 142 | 431 | √ |
| 1dtdB00 | 61 | 1 | √ | 2oojB00 | 134 | 362 | |
| 1dw9A02 | 68 | 396 | | 2ot9A01 | 176 | 636 | |
| 1dzfA01 | 138 | 516 | √ | 2oyrA01 | 55 | 18 | |
| 1dzfA02 | 73 | 608 | √ | 2p12A01 | 161 | 846 | |
| 1e0fI01 | 33 | 10 | √ | 2p5zX02 | 97 | 3936 | √ |
| 1e7lA01 | 80 | 655 | √ | 2p84A01 | 60 | 1 | √ |
| 1e7uA04 | 158 | 2556 | √ | 2ph0B00 | 162 | 1182 | |
| 1eg3A01 | 38 | 178 | √ | 2pk8A00 | 94 | 36 | √ |
| 1egwB01 | 61 | 3211 | √ | 2ppqA02 | 214 | 25795 | |
| 1ej6A01 | 383 | 22 | √ | 2prvB00 | 152 | 2796 | |
| 1ejgA00 | 46 | 115 | √ | 2q03B00 | 131 | 355 | |
| 1f0lA01 | 187 | 1 | √ | 2q83A02 | 233 | 21545 | |
| 1f3mA00 | 70 | 788 | √ | 2qgmA02 | 72 | 1029 | |
| 1g31A00 | 107 | 2287 | √ | 2qh9A00 | 178 | 319 | |
| 1g3pA02 | 104 | 2 | √ | 2qhqB00 | 120 | 400 | |
| 1gaxA04 | 50 | 1 | √ | 2qikA02 | 157 | 4229 | |
| 1gd8A00 | 105 | 2763 | √ | 2qnuA00 | 211 | 512 | |
| 1gqiA03 | 237 | 538 | √ | 2qyaA01 | 107 | 232 | √ |
| 1h16A00 | 759 | 2311 | | 2qziA00 | 101 | 150 | |
| 1h3iA01 | 134 | 16552 | √ | 2r01A02 | 42 | 245 | √ |
| 1h5wB03 | 45 | 18 | √ | 2r6iA01 | 96 | 1005 | |

| CATH Domain | *L* | *N* | In shrunk set | CATH Domain | *L* | *N* | In shrunk set |
|---|---|---|---|---|---|---|---|
| 1h6wA01 | 41 | 25 | √ | 2r6zA01 | 54 | 1 | √ |
| 1h8gA00 | 95 | 5753 | | 2ra8A01 | 74 | 1052 | √ |
| 1hbnA01 | 99 | 76 | √ | 2ra9A01 | 54 | 551 | √ |
| 1hh2P01 | 125 | 2627 | √ | 2ra9A02 | 73 | 798 | |
| 1hk8A00 | 561 | 2132 | √ | 2rciA00 | 188 | 104 | √ |
| 1hq0A00 | 295 | 42 | √ | 2re3A01 | 83 | 599 | |
| 1hx6A01 | 229 | 1 | √ | 2re3A02 | 70 | 793 | |
| 1i5pA03 | 198 | 27 | √ | 2rhqB03 | 202 | 4678 | √ |
| 1ihrA00 | 73 | 8185 | √ | 2rhqB04 | 75 | 4700 | √ |
| 1ikpA02 | 158 | 6 | √ | 2sicI00 | 107 | 424 | √ |
| 1io1A03 | 95 | 25 | √ | 2v0cA03 | 57 | 1 | √ |
| 1iq4A00 | 179 | 3389 | √ | 2v2gA02 | 70 | 3307 | √ |
| 1iq8A02 | 70 | 1 | √ | 2v3iA00 | 433 | 1398 | √ |
| 1iv8A02 | 118 | 1339 | | 2v9bA00 | 46 | 122 | √ |
| 1j5uA01 | 117 | 743 | | 2vl6A01 | 99 | 2447 | √ |
| 1jb0D00 | 138 | 142 | √ | 2vycA04 | 125 | 2191 | √ |
| 1jb7B00 | 216 | 9 | √ | 2w16A03 | 543 | 26498 | √ |
| 1jeyB02 | 201 | 2256 | √ | 2w86A02 | 60 | 91 | √ |
| 1jg5A00 | 83 | 76 | √ | 2wbmA01 | 81 | 831 | √ |
| 1jhnA02 | 146 | 803 | √ | 2wnyA00 | 137 | 430 | √ |
| 1ji6A03 | 199 | 213 | √ | 2wxfA04 | 152 | 2189 | √ |
| 1jidA00 | 114 | 717 | √ | 2x1lC02 | 77 | 7828 | √ |
| 1js8B02 | 105 | 144 | √ | 2x5yA00 | 171 | 2965 | √ |
| 1k0rA01 | 99 | 2393 | √ | 2xc1A01 | 104 | 73 | √ |
| 1k90A02 | 159 | 58 | √ | 2xqhA01 | 135 | 1809 | √ |
| 1kb2B00 | 85 | 2488 | √ | 2yyvB00 | 224 | 905 | √ |
| 1kfiA02 | 109 | 7899 | √ | 2zxeA04 | 211 | 15438 | √ |
| 1kfiA03 | 126 | 7516 | √ | 3a1cA02 | 117 | 15328 | √ |
| 1kl7A01 | 93 | 2574 | | 3a6mA02 | 57 | 4240 | √ |
| 1kmoA01 | 128 | 20235 | √ | 3a9uA03 | 75 | 26251 | √ |
| 1kmoA02 | 519 | 26061 | √ | 3afcA02 | 54 | 1188 | √ |
| 1kskA02 | 167 | 17897 | | 3al9A02 | 54 | 1057 | √ |
| 1kvdB00 | 77 | 2 | √ | 3apaA00 | 138 | 2183 | √ |
| 1l1lA02 | 112 | 1716 | √ | 3ar4A04 | 244 | 15576 | √ |
| 1l5jA04 | 128 | 3803 | √ | 3b2vA01 | 66 | 189 | √ |
| 1l5jA05 | 180 | 5942 | √ | 3b8cA04 | 148 | 14469 | √ |
| 1l6rA02 | 64 | 212 | √ | 3b8pB00 | 174 | 366 | |
| 1l8nA03 | 205 | 538 | √ | 3bgyA00 | 226 | 20 | √ |
| 1latA00 | 71 | 2516 | √ | 3bl4A01 | 72 | 228 | |
| 1lmlA02 | 124 | 890 | √ | 3bvxA01 | 382 | 2670 | √ |
| 1lngA00 | 87 | 673 | √ | 3bxjA01 | 34 | 3 | √ |
| 1lpbA00 | 85 | 255 | √ | 3c2qA01 | 88 | 309 | |
| 1lshA01 | 263 | 972 | √ | 3c8iA00 | 127 | 93 | √ |
| 1lshA03 | 98 | 315 | √ | 3c8wA01 | 232 | 1528 | |
| 1lshA04 | 251 | 339 | √ | 3c9qA00 | 195 | 278 | |

| CATH Domain | $L$ | $N$ | In shrunk set | CATH Domain | $L$ | $N$ | In shrunk set |
|---|---|---|---|---|---|---|---|
| 1ltlA01 | 90 | 1787 | √ | 3ci0J01 | 104 | 617 | |
| 1m3yA01 | 188 | 296 | √ | 3cjrB01 | 70 | 2009 | √ |
| 1m3yA02 | 215 | 204 | √ | 3claA00 | 213 | 4739 | √ |
| 1m6bA04 | 100 | 2743 | √ | 3clqA04 | 168 | 237 | |
| 1m6bB04 | 98 | 2699 | √ | 3cmbA00 | 265 | 933 | |
| 1mdbA03 | 76 | 26054 | √ | 3cngA01 | 34 | 2185 | √ |
| 1mnmA01 | 72 | 2941 | √ | 3cniA00 | 145 | 7008 | |
| 1musA02 | 276 | 962 | √ | 3cryA00 | 169 | 4606 | √ |
| 1mwpA00 | 96 | 134 | √ | 3cslA01 | 128 | 20280 | √ |
| 1n0uA03 | 107 | 1105 | √ | 3cslA02 | 625 | 25808 | √ |
| 1n7zA02 | 158 | 55 | | 3csvA02 | 240 | 28498 | |
| 1nc7A00 | 116 | 126 | | 3cw9A03 | 80 | 26493 | √ |
| 1nltA02 | 66 | 5186 | √ | 3cwcA02 | 235 | 2645 | |
| 1nqeA01 | 124 | 20303 | √ | 3cxbA01 | 115 | 93 | √ |
| 1nt2B01 | 71 | 18 | √ | 3d37A02 | 85 | 682 | √ |
| 1nvpC00 | 47 | 262 | √ | 3d5pA00 | 133 | 3445 | |
| 1nvpD02 | 46 | 309 | √ | 3d7aA01 | 136 | 434 | √ |
| 1o22A00 | 149 | 2 | | 3dh3A02 | 181 | 18724 | √ |
| 1o7dA01 | 279 | 2519 | √ | 3dnhB02 | 81 | 923 | |
| 1o9iA02 | 63 | 518 | √ | 3do6A02 | 119 | 2546 | |
| 1oaoC03 | 176 | 257 | √ | 3do6A03 | 89 | 2303 | √ |
| 1oaoD05 | 130 | 208 | √ | 3dxqB02 | 211 | 22164 | |
| 1ojjA00 | 397 | 1277 | √ | 3eb7A03 | 197 | 231 | √ |
| 1okgA03 | 69 | 14 | | 3eqvA01 | 116 | 9098 | √ |
| 1olzA02 | 57 | 963 | √ | 3exmA01 | 195 | 1176 | √ |
| 1oqvA00 | 171 | 81 | √ | 3fhhA01 | 124 | 20921 | √ |
| 1ozjA00 | 126 | 351 | √ | 3fn2A00 | 97 | 14 | |
| 1p5dX01 | 146 | 7825 | | 3fwlA03 | 90 | 739 | |
| 1p5dX02 | 78 | 7907 | √ | 3fy6D01 | 109 | 35 | |
| 1p5dX03 | 119 | 7648 | √ | 3fylA00 | 74 | 2491 | √ |
| 1pinA01 | 32 | 4804 | √ | 3g2eB00 | 185 | 3813 | √ |
| 1pp0B00 | 194 | 104 | √ | 3g4nA01 | 89 | 38 | √ |
| 1pucA00 | 101 | 416 | √ | 3gasA01 | 79 | 1389 | |
| 1pv5A00 | 260 | 1046 | | 3h1dA02 | 80 | 3309 | √ |
| 1qakA01 | 79 | 4735 | √ | 3h1dA03 | 112 | 3376 | √ |
| 1qd1A01 | 180 | 599 | √ | 3hhtA00 | 202 | 668 | |
| 1qd5A00 | 257 | 690 | √ | 3hkvA00 | 192 | 2769 | √ |
| 1qgiA01 | 147 | 212 | √ | 3i3wA01 | 151 | 8098 | |
| 1qksA02 | 432 | 30257 | √ | 3i3wA03 | 82 | 6863 | |
| 1qmbB00 | 42 | 3761 | √ | 3ib5A00 | 342 | 402 | |
| 1qu3A05 | 92 | 2854 | √ | 3ic3A01 | 64 | 67 | |
| 1qw2A00 | 102 | 222 | | 3it4B01 | 79 | 2675 | √ |
| 1r0oB00 | 78 | 2518 | √ | 3iylW01 | 388 | 25 | √ |
| 1r3eA01 | 221 | 5056 | √ | 3j80j00 | 86 | 2129 | √ |
| 1r7lA00 | 103 | 143 | √ | 3jv1A00 | 182 | 817 | √ |

| CATH Domain | *L* | *N* | In shrunk set | CATH Domain | *L* | *N* | In shrunk set |
|---|---|---|---|---|---|---|---|
| 1r9dA01 | 779 | 2239 | | 3kepB00 | 148 | 615 | |
| 1rozA00 | 336 | 1108 | √ | 3ktwB00 | 93 | 673 | √ |
| 1rqgA02 | 154 | 12679 | √ | 3l4gB01 | 89 | 669 | √ |
| 1rv9A00 | 242 | 3723 | √ | 3l4gB03 | 74 | 4675 | √ |
| 1rzhH02 | 132 | 787 | √ | 3l60A01 | 220 | 6324 | √ |
| 1s2oA02 | 71 | 536 | √ | 3llzA00 | 133 | 1989 | √ |
| 1s7mA02 | 90 | 25 | √ | 3lp8A04 | 93 | 4429 | √ |
| 1sauA01 | 44 | 1005 | √ | 3maeA00 | 234 | 6406 | √ |
| 1sczA00 | 233 | 6403 | √ | 3mjfA04 | 96 | 4420 | √ |
| 1sg2B00 | 109 | 2576 | √ | 3mu6A01 | 60 | 3135 | √ |
| 1sr8A01 | 39 | 37 | √ | 3mudA01 | 119 | 113 | √ |
| 1sr8A02 | 153 | 1125 | √ | 3mzfA02 | 92 | 3729 | √ |
| 1sva400 | 331 | 101 | √ | 3mzwA04 | 87 | 2064 | √ |
| 1t3cA00 | 411 | 64 | √ | 3n75A04 | 117 | 2216 | √ |
| 1t8hA00 | 272 | 3774 | √ | 3na5A01 | 207 | 8458 | √ |
| 1td6A02 | 92 | 3 | √ | 3na5A03 | 125 | 7851 | √ |
| 1tf5A02 | 122 | 3194 | √ | 3ng9A00 | 520 | 234 | √ |
| 1tljB00 | 188 | 488 | √ | 3nvqA02 | 57 | 1159 | √ |
| 1to2I00 | 63 | 1336 | √ | 3nzmA00 | 163 | 965 | √ |
| 1tuoA01 | 148 | 7672 | √ | 3od8F00 | 90 | 913 | |
| 1tuoA02 | 85 | 7564 | √ | 3odaF00 | 90 | 922 | √ |
| 1tuoA03 | 117 | 7720 | √ | 3odcA00 | 93 | 870 | √ |
| 1twfB04 | 177 | 1468 | √ | 3og2A02 | 184 | 670 | √ |
| 1twfF00 | 84 | 527 | √ | 3og2A03 | 89 | 89 | √ |
| 1u2mA00 | 86 | 2272 | | 3or1C01 | 40 | 982 | √ |
| 1u8bA01 | 70 | 2838 | | 3os4A00 | 393 | 3672 | |
| 1u94A02 | 59 | 3448 | √ | 3ot2A00 | 179 | 7877 | |
| 1udxA01 | 154 | 3510 | √ | 3pfgA02 | 59 | 126 | √ |
| 1uunA02 | 52 | 60 | √ | 3pmgA01 | 197 | 7949 | |
| 1v30A00 | 118 | 2633 | √ | 3pmgA03 | 100 | 6441 | √ |
| 1v9kA00 | 227 | 19051 | | 3q0iA02 | 105 | 5515 | |
| 1vb3A01 | 81 | 2544 | √ | 3q7eA02 | 177 | 2229 | √ |
| 1vbkA02 | 82 | 2266 | √ | 3q9oA02 | 167 | 6 | √ |
| 1vbwA00 | 68 | 1162 | √ | 3qjoA02 | 112 | 153 | √ |
| 1vclA03 | 148 | 42 | √ | 3r0qA02 | 208 | 1931 | √ |
| 1vk1A02 | 130 | 109 | | 3rk1B02 | 89 | 1308 | |
| 1vkmA00 | 291 | 1155 | | 3rlfF03 | 88 | 441 | √ |
| 1vlpD00 | 416 | 3692 | | 3s6lD00 | 158 | 2929 | √ |
| 1vmoA00 | 163 | 509 | √ | 3smjA00 | 188 | 2678 | √ |
| 1vr7A00 | 120 | 1257 | | 3smtA02 | 164 | 1193 | |
| 1vraB01 | 85 | 2744 | | 3tc5A01 | 31 | 4713 | √ |
| 1w3fA02 | 165 | 609 | √ | 3tewA01 | 244 | 2071 | √ |
| 1w6sA00 | 595 | 15658 | √ | 3tm0A02 | 173 | 24737 | √ |
| 1w96A02 | 45 | 462 | √ | 3u7uA04 | 57 | 294 | √ |
| 1w99A03 | 180 | 219 | √ | 3v7nA01 | 95 | 2574 | √ |

| CATH Domain | $L$ | $N$ | In shrunk set | CATH Domain | $L$ | $N$ | In shrunk set |
|---|---|---|---|---|---|---|---|
| 1wdjA00 | 186 | 7945 | √ | 3v8xA01 | 132 | 20172 | √ |
| 1wlgA02 | 140 | 1888 | √ | 3vd6C02 | 45 | 2059 | √ |
| 1wn9A00 | 126 | 17 | | 3vorA00 | 182 | 41 | √ |
| 1wpuA00 | 147 | 212 | √ | 3vrdB03 | 74 | 423 | |
| 1wqaA01 | 152 | 8110 | √ | 3vsjA00 | 270 | 4915 | √ |
| 1wr8A02 | 69 | 1 | √ | 3vsjD00 | 299 | 4935 | √ |
| 1wruA02 | 88 | 1039 | √ | 3w5mA06 | 100 | 2526 | √ |
| 1wteA02 | 124 | 91 | √ | 3w6gA02 | 71 | 3368 | √ |
| 1wv8A00 | 71 | 405 | | 3w9eC00 | 229 | 40 | √ |
| 1x0cA01 | 171 | 112 | √ | 3wocA00 | 138 | 2010 | √ |
| 1x9yA01 | 170 | 20 | √ | 4a5sA02 | 443 | 22149 | √ |
| 1xfdA01 | 464 | 20666 | √ | 4aghA00 | 80 | 681 | √ |
| 1xfjA00 | 256 | 3753 | | 4aybB04 | 165 | 1491 | √ |
| 1xkwA01 | 100 | 20263 | √ | 4aybK00 | 84 | 469 | √ |
| 1xp8A02 | 60 | 3385 | √ | 4b7oA01 | 111 | 19751 | √ |
| 1ya5T01 | 84 | 39 | √ | 4bsgA02 | 216 | 262 | √ |
| 1yrtA02 | 144 | 62 | √ | 4c2mB04 | 182 | 1438 | √ |
| 1yvuA02 | 93 | 1 | | 4cu4A01 | 141 | 20448 | √ |
| 1ywmA01 | 98 | 13 | √ | 4d6gA02 | 94 | 26 | √ |
| 1z1yA01 | 170 | 23387 | | 4deqA02 | 53 | 57 | √ |
| 1zd0A01 | 130 | 587 | √ | 4dw1A02 | 274 | 402 | √ |
| 2a1kA00 | 215 | 97 | √ | 4e2uA00 | 168 | 2234 | √ |
| 2a50B00 | 167 | 164 | √ | 4edgA01 | 134 | 4686 | √ |
| 2a6hC03 | 180 | 2560 | √ | 4fevA02 | 172 | 22694 | √ |
| 2a6hC04 | 65 | 4018 | √ | 4fwwA02 | 46 | 201 | √ |
| 2ahxB02 | 111 | 1788 | √ | 4g0aA01 | 144 | 24 | √ |
| 2ahxB04 | 138 | 4490 | √ | 4g22A02 | 211 | 5121 | |
| 2aj7A00 | 157 | 855 | | 4g22B01 | 206 | 4796 | |
| 2arzA02 | 88 | 1346 | √ | 4gimC00 | 305 | 1155 | √ |
| 2atzA00 | 176 | 50 | | 4gipD02 | 89 | 68 | √ |
| 2au3A02 | 127 | 4712 | √ | 4gv2A02 | 210 | 3029 | √ |
| 2auwA01 | 82 | 806 | √ | 4h05A02 | 180 | 18052 | √ |
| 2b3yA02 | 128 | 7764 | √ | 4hc7A02 | 60 | 2281 | √ |
| 2b5dX01 | 404 | 2684 | √ | 4hc9A02 | 52 | 2206 | √ |
| 2b5uA03 | 98 | 142 | √ | 4hjhA02 | 81 | 7580 | √ |
| 2b97A00 | 70 | 243 | √ | 4hn5A00 | 72 | 2472 | √ |
| 2bghA01 | 207 | 4903 | √ | 4ikpA02 | 188 | 2246 | √ |
| 2blnA02 | 102 | 5870 | √ | 4il3A02 | 84 | 10411 | √ |
| 2bmlA00 | 125 | 7086 | √ | 4ip2A02 | 129 | 1437 | √ |
| 2bpa100 | 426 | 368 | √ | 4iqfA02 | 105 | 5927 | |
| 2bw0A02 | 102 | 5665 | √ | 4issA03 | 137 | 1344 | |
| 2c42A03 | 212 | 5756 | √ | 4iyaA00 | 283 | 76 | √ |
| 2cn4A01 | 127 | 95 | √ | 4jimA03 | 90 | 2782 | √ |
| 2cxiA01 | 79 | 721 | √ | 4k3jB02 | 49 | 197 | √ |
| 2cxiA03 | 70 | 4719 | | 4kl5B00 | 141 | 1237 | √ |

| CATH Domain | *L* | *N* | In shrunk set | CATH Domain | *L* | *N* | In shrunk set |
|---|---|---|---|---|---|---|---|
| 2d5bA02 | 111 | 12119 | √ | 4kroA04 | 91 | 2020 | √ |
| 2e1vA01 | 233 | 4806 | | 4krpA03 | 67 | 1275 | √ |
| 2f20B00 | 232 | 3928 | | 4lgtA02 | 190 | 18306 | |
| 2f6eA00 | 125 | 7648 | √ | 4mbyB00 | 275 | 107 | √ |
| 2fdoA00 | 93 | 3 | | 4me3A01 | 94 | 512 | √ |
| 2fpnA01 | 140 | 109 | | 4mj0D00 | 267 | 105 | √ |
| 2fuvA02 | 95 | 7764 | | 4mq0A02 | 145 | 2083 | √ |
| 2fytA02 | 170 | 2263 | √ | 4n72B00 | 245 | 6499 | √ |
| 2fyuI00 | 57 | 136 | √ | 4o1rA00 | 142 | 2143 | √ |
| 2g16B00 | 163 | 163 | √ | 4o5nA01 | 216 | 277 | √ |
| 2g3wA00 | 179 | 640 | | 4o6uA00 | 182 | 96 | √ |
| 2g7cB03 | 97 | 6621 | √ | 4oltA02 | 95 | 239 | √ |
| 2gagD00 | 91 | 748 | √ | 4p3hA00 | 190 | 62 | √ |
| 2gjvA00 | 136 | 87 | √ | 4p72A03 | 199 | 5216 | √ |
| 2gkpA00 | 163 | 135 | | 4pchB00 | 266 | 107 | √ |
| 2glfA02 | 135 | 2091 | | 4pk9A00 | 359 | 11502 | √ |
| 2gskA02 | 464 | 25890 | √ | 4q7gA00 | 300 | 94 | √ |
| 2gu0A01 | 142 | 22 | √ | 4rreC01 | 35 | 4337 | √ |
| 2gukA00 | 111 | 135 | | 4uwhA02 | 154 | 2763 | √ |
| 2gy5A03 | 135 | 19253 | √ | 4uykA00 | 81 | 320 | |
| 2h21A02 | 165 | 1227 | √ | 4wz8B02 | 66 | 547 | √ |
| 2h21C01 | 254 | 3144 | √ | 7ahlF01 | 292 | 72 | √ |
| 2hdiB00 | 103 | 3 | √ | | | | |

*L* is the chain length while *N* is the number of sequences in the MSA. The shrunk set was used to train models in pipeline with RaptorX-Contact.

**Table S2. Results of structure prediction for 61 mainly β proteins.**

| Name | β residue proportion | TM-score | | RMSD (Å) | | Number of $RDb_2C$ Predictions | Number of β contacts |
|---|---|---|---|---|---|---|---|
| | | RaptorX-Contact | $RDb_2C$ | RaptorX-Contact | $RDb_2C$ | | |
| 1alya | 52.05% | 0.5070 | **0.5332** | 12.65 | **7.55** | 0.315L | 0.336L |
| 1amxa | 54.00% | 0.5690 | **0.6410** | 14.33 | **5.06** | 0.313L | 0.353L |
| 1aoha | 57.34% | 0.5239 | **0.6210** | 7.23 | **4.08** | 0.294L | 0.371L |
| 1azpa | 53.03% | **0.3504** | 0.3075 | 12.65 | **8.16** | 0.167L | 0.288L |
| 1bdya | 53.66% | **0.5524** | 0.5454 | 10.23 | **8.66** | 0.301L | 0.341L |
| 1bkza | 54.81% | 0.4075 | **0.6917** | 7.05 | **3.78** | 0.378L | 0.370L |
| 1bnda | 60.55% | 0.3768 | **0.4587** | 8.13 | **6.68** | 0.239L | 0.312L |
| 1c3ha | 53.28% | 0.6467 | **0.6597** | 4.28 | **3.99** | 0.372L | 0.336L |
| 1c9sm | 59.15% | **0.3972** | 0.3845 | 9.44 | **6.69** | 0.254L | 0.366L |
| 1dqta | 56.41% | 0.5936 | **0.6315** | 6.67 | **4.51** | 0.282L | 0.376L |
| 1eaja | 50.00% | 0.5422 | **0.6490** | 10.71 | **4.47** | 0.355L | 0.339L |
| 1fhga | 59.80% | 0.5920 | **0.6674** | 6.54 | **4.32** | 0.333L | 0.353L |
| 1flma | 50.00% | 0.6231 | **0.6767** | 4.25 | **3.86** | 0.311L | 0.311L |
| 1fnla | 52.60% | 0.3370 | **0.3400** | **15.35** | 18.77 | 0.324L | 0.295L |
| 1fzva | 56.00% | 0.3414 | **0.3753** | 10.68 | **9.66** | 0.280L | 0.270L |
| 1g43a | 54.37% | 0.5071 | **0.6263** | 8.41 | **5.98** | 0.319L | 0.331L |
| 1gc1c | 50.28% | 0.2914 | **0.3944** | 20.75 | **15.66** | 0.271L | 0.309L |
| 1gwma | 50.33% | 0.5186 | **0.5249** | **9.96** | 11.29 | 0.268L | 0.327L |
| 1h5ba | 53.10% | 0.5778 | **0.6068** | 5.57 | **4.21** | 0.345L | 0.354L |
| 1h64k | 56.34% | 0.4742 | **0.5863** | 5.08 | **3.10** | 0.324L | 0.366L |
| 1h6xa | 51.57% | 0.5131 | **0.5615** | 7.18 | **6.15** | 0.352L | 0.321L |
| 1h9ka | 50.00% | 0.2314 | **0.4305** | 14.51 | **6.52** | 0.319L | 0.319L |
| 1hkfa | 55.56% | 0.5537 | **0.6532** | 6.14 | **4.62** | 0.333L | 0.361L |
| 1hzaa | 55.22% | 0.4198 | **0.5591** | 7.17 | **4.97** | 0.299L | 0.313L |
| 1ibya | 66.07% | 0.3708 | **0.4435** | **19.61** | 21.13 | 0.295L | 0.375L |
| 1ifga | 52.14% | 0.3855 | **0.3924** | 10.98 | **8.20** | 0.229L | 0.293L |
| 1igub | 53.33% | 0.3744 | **0.4979** | 10.85 | **5.07** | 0.283L | 0.300L |
| 1im3d | 63.16% | 0.3106 | **0.3197** | 20.21 | **15.74** | 0.179L | 0.411L |
| 1iz6a | 54.41% | 0.3105 | **0.4896** | 14.94 | **5.99** | 0.360L | 0.331L |
| 1j48a | 55.05% | **0.3038** | 0.2906 | 12.44 | **10.84** | 0.312L | 0.330L |
| 1jsga | 51.35% | 0.2247 | **0.2581** | 19.87 | **18.12** | 0.072L | 0.351L |
| 1jzdc | 65.25% | 0.5246 | **0.6288** | 6.43 | **5.39** | 0.297L | 0.381L |
| 1k4zb | 61.78% | 0.4571 | **0.5926** | 14.69 | **6.78** | 0.452L | 0.414L |
| 1kq1h | 50.00% | 0.4425 | **0.5665** | 5.56 | **4.14** | 0.273L | 0.303L |
| 1kqra | 56.25% | 0.2851 | **0.3406** | 14.73 | **12.52** | 0.281L | 0.356L |
| 1krla | 61.36% | **0.3931** | 0.2961 | 7.63 | **6.20** | 0.159L | 0.364L |
| 1kxgb | 54.86% | 0.4530 | **0.4760** | 25.11 | **16.70** | 0.243L | 0.368L |
| 1l5ba | 55.45% | 0.2902 | **0.3688** | **12.44** | 14.34 | 0.327L | 0.307L |
| 1lshb | 50.00% | **0.5370** | 0.4288 | **6.35** | 8.57 | 0.408L | 0.368L |
| 1lyqb | 50.00% | 0.4881 | **0.5042** | **5.78** | 6.26 | 0.221L | 0.327L |
| 1m8na | 59.17% | **0.2244** | 0.2149 | 14.62 | **14.56** | 0.000L | 0.467L |
| 1mbya | 53.33% | 0.3073 | **0.3316** | 14.87 | **13.72** | 0.267L | 0.320L |
| 1mm9a | 52.76% | 0.3189 | **0.3266** | 8.72 | **8.55** | 0.323L | 0.394L |

| Name | β residue proportion | TM-score | | RMSD (Å) | | Number of $RDb_2C$ Predictions | Number of β contacts |
|---|---|---|---|---|---|---|---|
| | | RaptorX-Contact | $RDb_2C$ | RaptorX-Contact | $RDb_2C$ | | |
| 1nepa | 54.62% | 0.3799 | **0.5315** | 8.29 | **5.50** | 0.331L | 0.354L |
| 1nezg | 50.82% | 0.5896 | **0.6330** | 6.38 | **4.20** | 0.303L | 0.344L |
| 1nlqa | 59.05% | 0.5649 | **0.6345** | 4.20 | **3.61** | 0.400L | 0.390L |
| 1npua | 56.03% | 0.5111 | **0.6089** | 6.82 | **4.41** | 0.353L | 0.371L |
| 1nyca | 54.95% | **0.3542** | 0.3372 | **14.62** | 15.14 | 0.126L | 0.369L |
| 1o5ua | 59.09% | **0.4727** | 0.4660 | **14.27** | 14.28 | 0.295L | 0.386L |
| 1oarh | 53.28% | 0.6024 | **0.6258** | 5.83 | **4.93** | 0.369L | 0.344L |
| 1ousb | 62.28% | 0.3612 | **0.6172** | 10.84 | **4.13** | 0.377L | 0.412L |
| 1pm4a | 52.99% | 0.3536 | **0.3584** | 9.39 | **9.00** | 0.205L | 0.325L |
| 1py9a | 52.59% | 0.5250 | **0.6425** | 9.49 | **4.58** | 0.276L | 0.336L |
| 1qu0c | 50.82% | 0.5608 | **0.5893** | 10.12 | **7.65** | 0.333L | 0.350L |
| 1rj8a | 58.57% | **0.2949** | 0.2744 | **15.09** | 16.80 | 0.243L | 0.379L |
| 1rlwa | 50.79% | 0.5844 | **0.6591** | 6.12 | **4.12** | 0.365L | 0.325L |
| 1sppb | 56.25% | 0.5805 | **0.6502** | 7.49 | **6.41** | 0.330L | 0.366L |
| 1tula | 50.98% | 0.3146 | **0.4330** | 10.74 | **6.96** | 0.235L | 0.324L |
| 1uxza | 61.83% | 0.4335 | **0.6257** | 8.57 | **4.02** | 0.374L | 0.382L |
| 1whoa | 50.00% | 0.5512 | **0.5923** | 6.44 | **3.96** | 0.351L | 0.298L |
| 1xcaa | 55.47% | 0.4912 | **0.7190** | 8.39 | **3.24** | 0.409L | 0.394L |
| | | Average 0.4422 | Average **0.5064** | Average 10.39 | Average **8.01** | Maximum 0.452L | Maximum 0.467L |

Winner in each category is highlighted in bold.

# Supplementary references

1. Lindeberg T, Edge detection and ridge detection with automatic scale selection. In: *Computer Vision and Pattern Recognition, 1996 Proceedings CVPR'96, 1996 IEEE Computer Society Conference on: 1996*. IEEE: 465-470.
